# Nanoscale Phase Change Material Array by Sub-Resolution Assist Feature for Storage Class Memory Application

**DOI:** 10.3390/nano13061050

**Published:** 2023-03-15

**Authors:** Jiarui Zhang, Wencheng Fang, Ruobing Wang, Chengxing Li, Jia Zheng, Xixi Zou, Sannian Song, Zhitang Song, Xilin Zhou

**Affiliations:** 1 State Key Laboratory of Functional Materials for Informatics, Shanghai Institute of Microsystem and Information Technology, Chinese Academy of Sciences, Shanghai 200050, China; 2 School of Physical Science and Technology, ShanghaiTech University, Shanghai 201210, China

**Keywords:** phase change memory, storage class memory, SRAF, optical proximity effect, micro-loading effect

## Abstract

High density phase change memory array requires both minimized critical dimension (CD) and maximized process window for the phase change material layer. High in-wafer uniformity of the nanoscale patterning of chalcogenides material is challenging given the optical proximity effect (OPE) in the lithography process and the micro-loading effect in the etching process. In this study, we demonstrate an approach to fabricate high density phase change material arrays with half-pitch down to around 70 nm by the co-optimization of lithography and plasma etching process. The focused-energy matrix was performed to improve the pattern process window of phase change material on a 12-inch wafer. A variety of patternings from an isolated line to a dense pitch line were investigated using immersion lithography system. The collapse of the edge line is observed due to the OPE induced shrinkage in linewidth, which is deteriorative as the patterning density increases. The sub-resolution assist feature (SRAF) was placed to increase the width of the lines at both edges of each patterning by taking advantage of the optical interference between the main features and the assistant features. The survival of the line at the edges is confirmed with around a 70 nm half-pitch feature in various arrays. A uniform etching profile across the pitch line pattern of phase change material was demonstrated in which the micro-loading effect and the plasma etching damage were significantly suppressed by co-optimizing the etching parameters. The results pave the way to achieve high density device arrays with improved uniformity and reliability for mass storage applications.

## 1. Introduction

Storage class memory (SCM) has been recognized as a promising candidate to break through the inherent limitations of current computational architecture due to its high density, low power, and high speed [1,2,3,4]. Phase change memory (PCM) utilizes the reversible switching from a highly resistive amorphous phase to a conductive crystalline phase in the data storage [5,6,7,8,9]. It is fascinating by virtue of the huge resistance window with up to three orders of magnitude of difference in resistivity that enables the requisite multi-level storage in SCM [10,11,12,13]. Geometrical scaling and low power per bit of the PCM arrays is crucial to addressing the SCM application. Both the minimized CD in three dimensions and the maximized tolerance of the process parameter of the phase change material are required to fulfill the demand for a high density PCM array on a large scale. However, the improvement of the uniformity on 12-inch wafers of the nanoscale patterning of the chalcogenides material is challenging for manufacturing due to the inherent OPE in the lithography process [14,15] and the micro-loading effect in the etching process [16,17].

**Figure 1:**
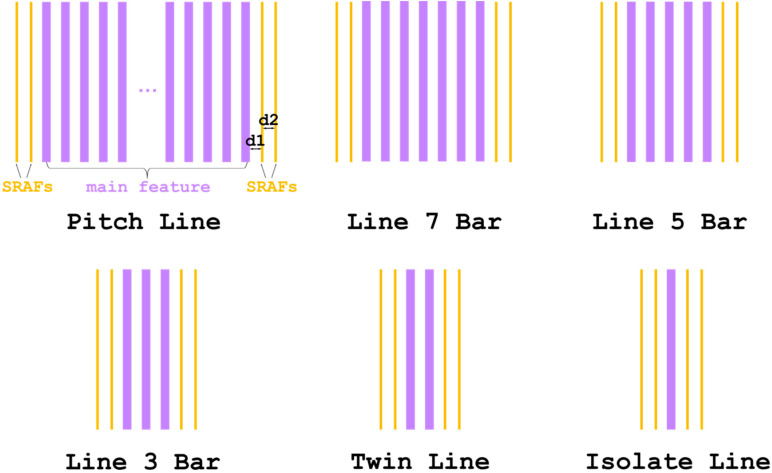
Schematic diagram of the various PCM arrays in the mask in the presence of SRAFs. The line number of the main feature varies from 57 in the pitch line to 1 in the isolate line. The main features are represented by the violet lines and the SRAFs are the yellow lines. *d*_1_ and *d*_2_ is the distances of the lines at the edge of main feature to the nearest SRAFs and the next nearest SRAFs, respectively.

There is a remarkable discrepancy, which increases as the critical dimension decreases, between the patternings designed in the mask and fabricated on the wafer due to the diffraction-limited property of the lithographic imaging systems [18]. Various resolution enhancement techniques are presented to reduce the OPE and improve the quality and stability of the lithographic patterning. The sub-resolution assist feature (SRAF) added adjacent to the main feature based on the design rule or model is one of the typical techniques used to enhance the process window of the lithography process [19]. The rule-based approach designs the SRAF’s layout by referring a table to decide the size, number, and relevant distances of the assist features according to the CD and pitch of the main feature [20,21], while the model-based approach is based on the computed result of simulation or inverse lithography technology [22,23]. In this study, the OPE-caused CD shrink is observed at the edges of various phase change material line arrays, irrespective of the line density of the array. To address the undesired effect the SRAF, which is composed of two identical lines and illustrated as the outermost narrow lines of the patterns in Figure 1, are inserted to both sides of the main features (the violet lines in Figure 1) of all arrays in the mask pattern. A two-line SRAF design is determined by Fourier optics, scalar diffraction theory, and source point integration [24,25,26], in which the final light intensity reached on the wafer is the incoherent sum of each source point’s contribution to the overall image. The light from a source point would diffract when it passes the mask. Then, the projection lens collects and focuses the diffracted lights with the spatial frequencies lower than the quotient of the numerical aperture divided by the wavelength of the light source in the lithography system to form the aerial image on the wafer. A single line SRAF has limited influence on the light intensity at the edge of the main feature and an SRAF design with more than two lines could not provide significant improvement in the performance yet occupies a larger area in the mask. The distances between the edges of main features and the nearest SRAF and the next nearest SRAF are defined as *d*_1_ and *d*_2_, respectively, and Δ*d* = *d*_1_ − *d*_2_. The effect of the distance from SRAFs to the edges of the main feature is investigated and the optimized SRAFs are employed to acquire the uniform arrays for the lithography process and the subsequent etching by effectively offsetting the OPE.

In the process of plasma etching, the etching rate in the region with dense patterns is much slower than in the region with sparse lines, which is known as the micro-loading effect [16,17,27]. The parameters in the etching process, such as the transformer coupled power and bias voltage, have been tuned carefully to eliminate the micro-loading effect and thus achieve uniform patterns with high fidelity [28,29,30]. Carbon-doped Ge_2_Sb_2_Te_5_ (GSTC) is one of the most promising phase change materials for the next generation nonvolatile data storage application due to its fast speed, high thermal stability, and excellent scalability [31,32,33,34,35,36]. In this study, we realize a uniform etching profile across the pitch line array based on the patterns fabricated by lithography with SRAF. The co-optimization of the lithography and etching process produce uniform 70 nm-wide line arrays composed of a TiN/GSTC phase change material film stack across the 12-inch wafer which is beneficial for the memory arrays with high density and high reliability for the storage class memory applications.

## 2. Experimental Details

**Film preparation.** A 300 nm-thick SiO_2_ film was deposited on a 12-inch silicon wafer by chemical vapor deposition at 350 °C. The 100 nm-thick GSTC material was then deposited on the SiO_2_ layer by sputtering at an ambient temperature using an alloy target which is followed by the deposition of TiN film of 40 nm in a reactive sputtering mode.

**Lithography process.** The photolithography was performed in an immersion lithography system with an argon fluoride light source [37]. The quad-layer stack using positive photoresist was successfully imaged and developed to form various line array patterns. Compared to the tri-layer scheme, a quad-layer stack could facilitate the control of the light intensity reflected to the bottom of photoresist [38]. In this work, the quad-layer stack consisted of a 166 nm-thick positive photoresist, a 70 nm-thick bottom anti-reflective coating, an 80 nm-thick SiO_2_ and a 200 nm-thick spin-on carbon (SOC) from top to bottom. The photoresist, bottom anti-reflective coating, and SOC films were prepared at ambient temperature and baked at 250, 205, and 115 °C, respectively, in different tools. The SiO_2_ layer, which acts as the hard mask of the etching, was prepared in a chemical vapor deposition system at 200 °C. The energy dose and focal plane position in the scanner were set as 26, 34, and 38 mJ/cm^2^ and 0 nm, respectively, under an annular light source. A post-exposure baking at 115 °C was conducted before a positive developing solution was applied.

**Etching process.** The gas mixture, including CF_2_ with 120 standard cubic centimeter per minute (sccm) and CH_2_F_2_ with 30 sccm gas, was utilized to etch the SiO_2_ layer at 6 mTorr for around 80 s. The SOC was etched for around 100 s by a mixed gas of 200 sccm Ar and 36 sccm O_2_ at a chamber pressure of 5 mTorr. The 40 sccm BCl_3_ and 60 sccm Cl_2_ gas mixture was used in the TiN film etching at 3 mTorr for 20 s. For the etching of GSTC phase change material, an HBr and He mixture gas, of which the component flow rates were 120 and 300 sccm, respectively, was adopted for 50 s in an inductive coupled plasma system under a chamber pressure of 5 mTorr. Finally, the residual materials of the quad-layer stack were fully removed by a strip step for 50 s at 15 mTorr. The mixture gas consisted of 100 sccm N_2_ and 80 sccm O_2_.

**Characterization.** The nanoscale morphology of the patterned line arrays was analyzed using a scanning electron microscopy (SEM) system at an accelerate voltage of 500 V. The cross-sectional etching profile of the TiN/GSTC phase change material stack was observed using transmission electron microscopy (TEM) at 200 kV. The TEM sample was prepared using a focus ion beam (FIB). The carbon and platinum materials were deposited sequentially in the FIB system as capping layers to protect the patterned structure from the irradiation damage of the ions. Then, a V-shape pit was scooped along both sides of the film by ion beam. The sample was subsequently trimmed carefully into a U-shape. Additional platinum material was deposited between the micromanipulator tip and the sample to transfer the sample out from the substrate of the wafer using the micromanipulator tip. After the sample was moved to the TEM holder, additional ion beam milling was performed to reduce the sample thickness to around 50 nm. The elemental distribution was analyzed by the energy dispersive spectroscopy (EDS).

## 3. Results and Discussion

**Figure 2:**
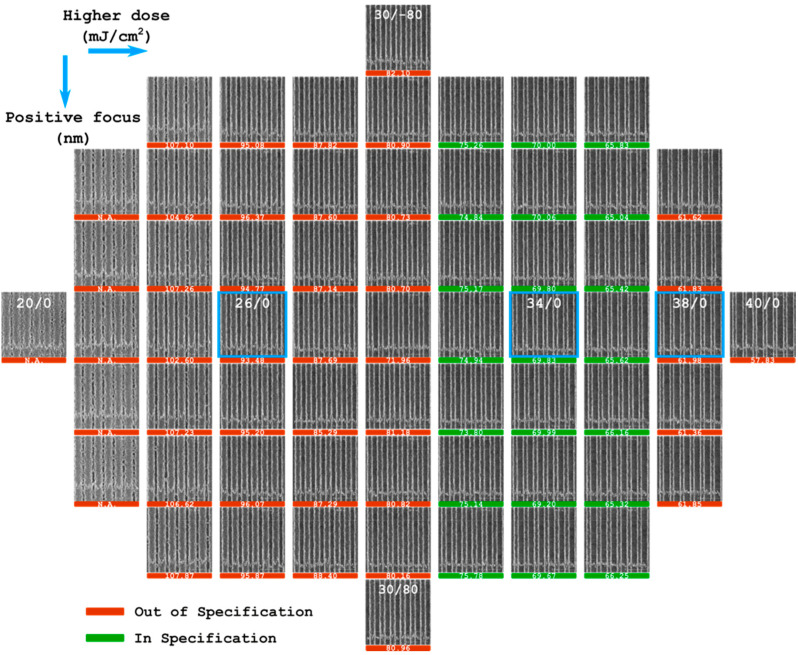
The experimental results of focused-energy matrix analysis after the developing process. Each shot on the wafer has a unique energy dose and focus position (E/F) which increases from left to right by 2 mJ/cm^2^ in energy dose and from up to down by 20 nm in focus position. The dose (E) is in the range of 20 mJ/cm^2^ to 40 mJ/cm^2^ and the focus (F) is in the range of −80 nm to 80 nm. The SEM images are acquired on the center of pitch line arrays of these shots. The E/F used in the shots are labeled on parts of the images. The CDs of the center lines in each shot are labeled under the corresponding images in nm. For the patterns with pronounced defects, the reliable CD measurement is not available, therefore N.A. is used as the label. The selected E/F parameters for the research in this work are marked with blue squares.

The process window for a 70 nm-wide pitch line at the center of the arrays shown in Figure 2 was obtained by performing the focused-energy matrix analysis, which suggests that the depth of focus (DOF) is larger than 120 nm and the energy latitude (EL) is around 14.7%. The exposure latitude is defined as the energy dose range which could meet the CD requirement in the process window [39,40]. The computing method is provided as EL = (E_max_ − E_min_)/E_nominal_ × 100%, where E_max_, E_min_, and E_nominal_ represent the energy doses corresponding to the CDs that are 10% less than, 10% more than, and equal to the target dimension under the nominal focus, respectively. According to the FEM result shown in Figure 2, we fitted the curve of the CD as a function of the energy dose under the 0 nm focus which is a straight line, CD = −2.757E + 165.66, where E is the energy dose of the light source. E_max_ and E_min_ is calculated to be 37.2 and 32.2 mJ/cm^2^, respectively. E_nominal_ equals to 34 mJ/cm^2^ which is the optimized energy dose as given in Figure 2. As a result, the energy latitude is determined to be 14.7%. The sufficient process window demonstrates that the quad-layer stack consisting of photoresist, anti-reflective coating, SiO_2_, and spin-on carbon together with the annular illumination in the lithography configuration provide a reliable lithography process in manufacturing the PCM line arrays with a half-pitch feature down to around 70 nm. The energy dose of 34 mJ/cm^2^ and focus distance of 0 nm were selected as the optimal conditions for the PCM arrays as demonstrated in Figure 2. The lithography morphology of the arrays under 26 and 38 mJ/cm^2^ were also recorded to investigate the effect of the energy dose of the light source.

**Figure 3:**
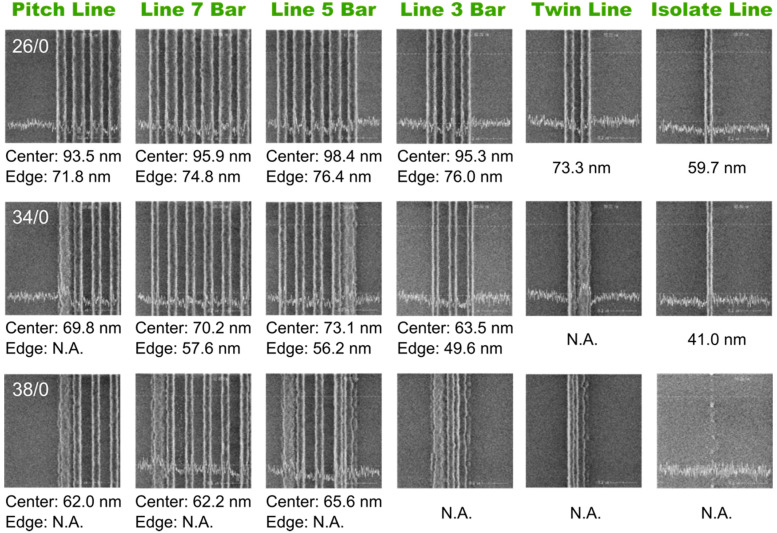
The lines containing only photoresist represent the pattern of the photomask. The lithography is performed without adding SRAF in the mask. The array types are labeled on the top of figure and the selected E/F parameters of 26/0, 34/0, and 38/0 are label on the pitch line image of the top, middle, and bottom panel, respectively. The CDs of lines at the center and edge of array are labeled under the images. The CDs of collapsed lines are labeled as N.A. Single CD value is labeled under each image of the twin line and isolate line arrays.

Figure 3 shows the SEM images of various line arrays patterned by immersion lithography at a fixed focus position of 0 nm and different energy doses of 26, 34, and 38 mJ/cm^2^, respectively, from top to bottom panels. The line dimension at the center of each array is decreased as the dose increases. A line width of around 70 nm is observed at the center of the pitch line, line 7 bar, and line 5 bar arrays under an energy of 34 mJ/cm^2^, in which the failure of the edge line is present in the pitch line, line 5 bar, and twin line. What is more, the collapse of the lines at the edge of the array is confirmed in all arrays under an energy of 38 mJ/cm^2^, indicating a strong non-uniformity patterning induced by both the OPE and the energy dose. The thinner line patterns at the edges are not survived due to the considerable surface tension in the developing process. It is noted that the line patterns of the twin line and isolate line also exhibit the shrinkage in width due to the similar adjacent environment to the edges of denser line arrays. That is, they are always at the edge of the arrays since there is no nominal center line. However, interestingly, the isolate line seems to be less affected by the surface tension and the pattern has survived the lithography process with the width down to 41 nm. As the energy dose decreases to 26 mJ/cm^2^, the line width at both the center and edge of all the arrays increases significantly as shown in the top panel of Figure 3, in which all the line patterns are preserved after developing and OPE-induced shrinkage in the line width at the edge of the arrays is observed.

The OPE found in Figure 3 is caused by the discrepancy of the local environment of the pattern. The finite collection capability of the diffracted light of the pupil in the lithography system leads to the fact that the mutual interference of adjacent patterns on the mask could not be compensated by the diffracted light in high orders. The light intensity at the edges of the array is affected by the interference light from both the space side and the line array side, while the intensity at the array centers is affected by the light from the lines on both sides. The higher intensity magnitude of the light from the space side gives rise to the shrink of the lines at the edge by promoting the photochemical reaction. It is noted from Figure 3 that the lines adjacent to the line at edge are preserved in all experimental cases, suggesting a weaker shrink in size or a wider line than the edge one. This is because the interference light of the space is partially shielded by the lines at the edge which improve the uniformity of the light intensity on both sides of the line next to the edge one.

**Figure 4:**
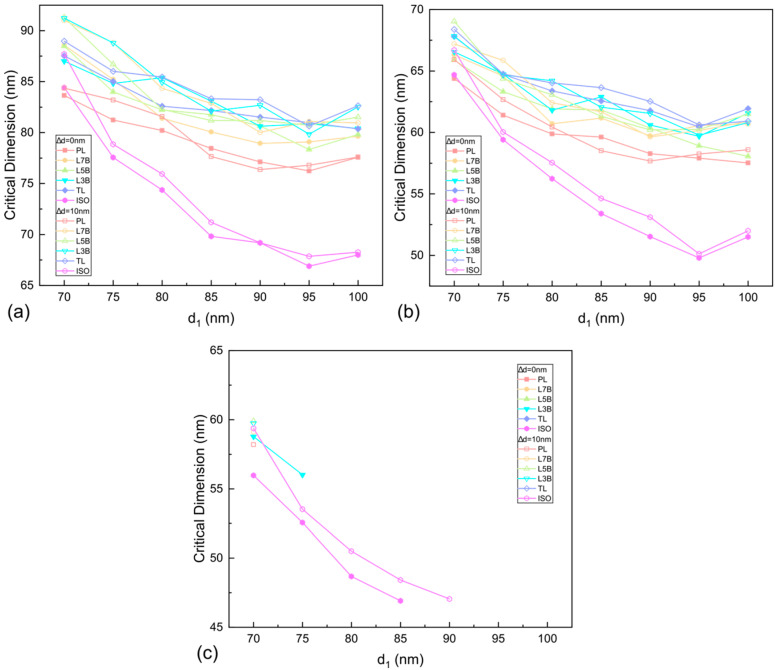
The CD of the line at the edges of various of patterns as a function of *d*_1_ under an energy dose of (**a**) 26 mJ/cm^2^, (**b**) 34 mJ/cm^2^, and (**c**) 38 mJ/cm^2^. The focus position is kept at 0 nm. The SRAF is classified by ∆*d* = *d*_1_ − *d*_2_, which is 0 or 10 nm.

The insertion of SRAF in the mask is an essential method by which to remove the OPE, as it acts like an extra line outside the edge of the pattern to effectively shield the interference light of the space area which would not be printed on the photoresist. SRAF has been placed on the sides of the isolate and semi-isolate patterns to enlarge the process window patterns [41]. As shown above in Figure 1, two assist features are added to the edge of the main feature whose adjacent environments are thus similar to the isolate or semi-isolate lines. The width of the assist line is fixed at 25 nm, which is well beyond the resolution of the lithography system. The distance between the edge of arrays and the closest SRAF, *d*_1_, is in the range of 70 to 100 nm, while the spacing between the two assist features, *d*_2_, is determined by Δ*d*, where Δ*d* = 0 nm and Δ*d* = 10 nm are employed in this work. Figure 4 shows the critical dimension of the edge line of all the arrays as a function of SRAF configuration in different energy doses. The largest critical dimension of the edge line is found at *d*_1_ = 70 nm irrespective of the energy dose. It is substantially larger than that of the arrays in the cases without the presence of SRAFs shown in Figure 3, indicating that the OPE is well compensated by including the assist features. For the energy doses of 26 and 34 mJ/cm^2^ given in Figure 4a,b, the width of the line at the edge of arrays shows a decrease as the distance, *d*_1_, increases, in which the size of the edge line tends to get larger under the condition of Δ*d* = 10 nm. The line width around 70 nm of all the arrays is found at the energy dose of 34 mJ/cm^2^ with *d*_1_ = 70 nm and Δ*d* = 10 nm. It is worth noting that there is a more remarkable variation in the critical dimension of the isolate line as *d*_1_ increases since the SRAFs are inserted on both sides of the isolate line. As the energy E increases to 38 mJ/cm^2^, the edge lines are survived only under limited SRAF configurations as shown in Figure 4c, in which the minimum *d*_1_ of 70 nm is effective at modulating the interference light intensity for a wider line at the edge of the array. It is thus rational to adopt an SRAF design with a smaller *d*_1_ and *d*_2_, yet a larger width, to improve the fidelity of the line at the edge of the array which, on the other hand, could increase the risk of unfavorable SRAF print on the wafer.

**Figure 5:**
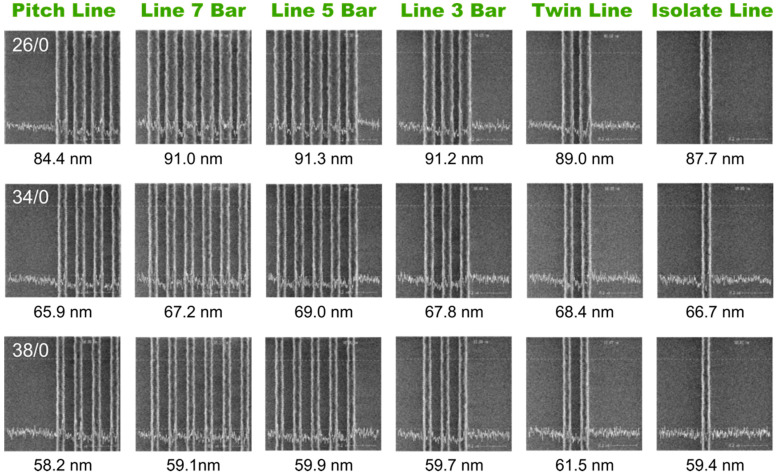
The SEM images focusing on the lines at the edge of various arrays after the developing process. The lithography is performed with additional SRAFs on the edge of the main feature in the mask. The array types are labeled on the top of figure and the selected E/F parameters of 26/0, 34/0, and 38/0 are labeled on the pitch line image of the top, middle, and bottom panels, respectively. The CDs of lines at the edge of the array are provided under the images.

**Figure 6:**
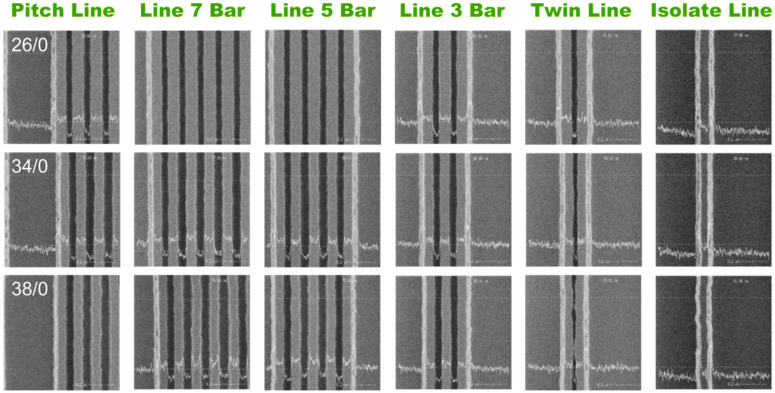
The SEM images focusing on the TiN/GSTC stack lines at the edge of various of arrays after the SRAF-lithography and subsequent plasma etching. The array types are labeled at the top of figure and the selected E/F parameters of 26/0, 34/0, and 38/0 are labeled on the pitch line image of the top, middle, and bottom panels, respectively. The white stripes at the edge are due to the over etch of SiO_2_ caused by the micro-loading effect.

The SEM results of various line arrays shown in Figure 5 are patterned with the optimized SRAF configuration of *d*_1_ = 70 nm and Δ*d* = 10 nm. The same lithography parameter is used as mentioned in Figure 3, where the energy dose is 26, 34, and 38 mJ/cm^2^, respectively, from top to bottom panels, and the focus position is 0 nm. It is found that the edge lines are completely preserved in all arrays with a considerably increased width, which is attributed to the decreased OPE at the edges. The SEM images also demonstrate the significantly improved uniformity in the line width both within array and inter-arrays, in which no fatal defects are detected in the patterns. What is more, the pattern feature is well preserved in the TiN/GSTC phase change material stack structure in all arrays after the optimized plasma etching process, as shown in Figure 6, in which the etching process transfers the photoresist patterns by SRAF first to the hard mask and then to the deposited film stack. Because of the sufficient etching selection ratio between the SOC and TiN film, the TiN film is almost intact after the SOC etching process, which indicates that the micro-loading effect is minimized in the SOC etching. Therefore, the micro-loading effect generated by the TiN etching process cannot be neglected. To transfer the optimized pattern of the photoresist by SRAF to the TiN/GSTC film stack, we reduced the transformer coupled power to 500 W and increased the bias voltage to 200 V in the TiN etching process. The optimized etching parameters successfully suppress the micro-loading effect and the associated plasma etching damage. The white stripes are identified in Figure 6 at both edges of all the arrays after etching. This is attributed to the over etching of the SiO_2_ layer outside the arrays, which is a typical behavior caused by the micro-loading effect of etching.

**Figure 7:**
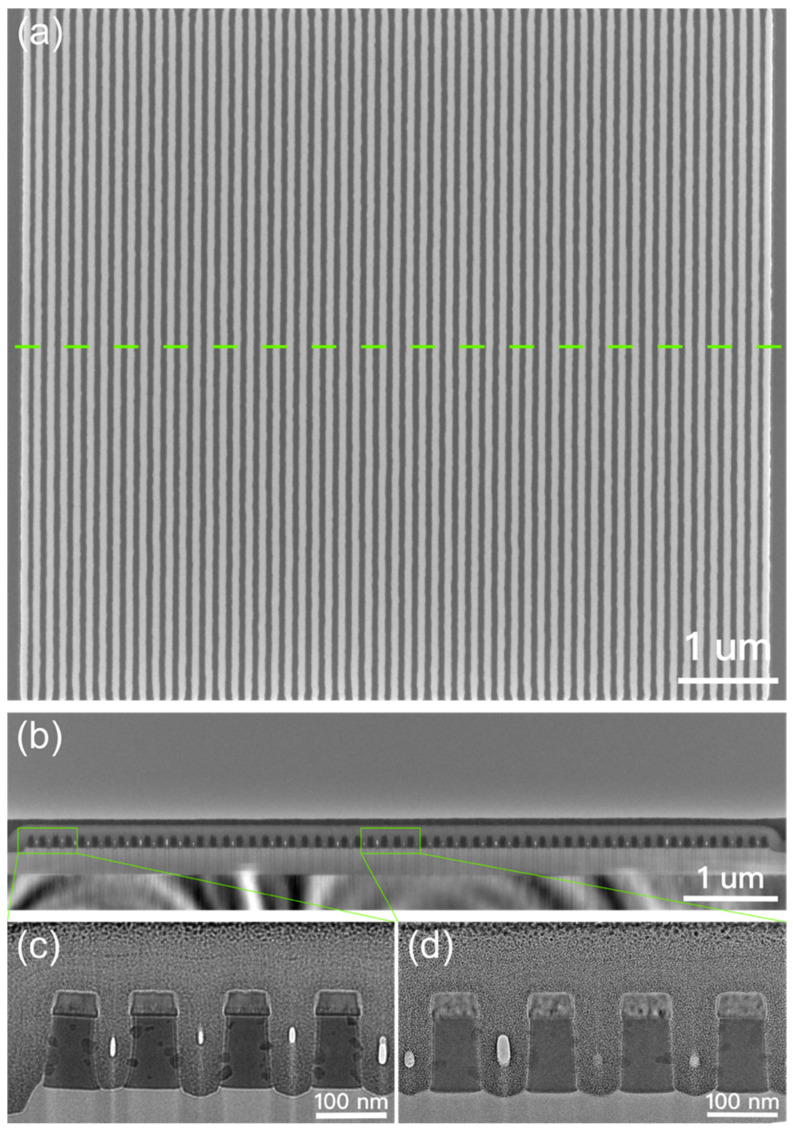
(**a**) The top view SEM image of the pitch line array composed of TiN/GSTC film stack after the SRAF-lithography and subsequent plasma etching. The E/F parameter is 34/0. (**b**) The cross-sectional TEM image of the pitch line array. The observed region is marked by the dashed line in green in (**a**). The magnified cross-sectional TEM image of the (**c**) edge and (**d**) center region of the pitch line array. The C and Pt films are deposited on the top of the stacked structure during the preparation of the TEM samples using the focused ion beam system.

**Figure 8:**
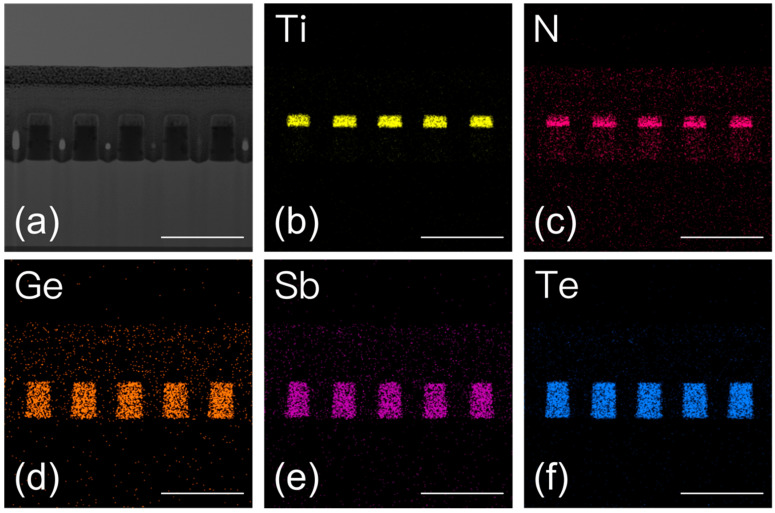
The STEM and corresponding elemental mapping analysis of the cross-sectional TiN/GSTC film stack after the SRAF-lithography and subsequent plasma etching: (**a**) STEM image and EDS map for (**b**) Ti, (**c**) N, (**d**) Ge, (**e**) Sb, and (**f**) Te. The scale bar is 200 nm. The C and Pt films are deposited on the top of the stacked structure during the preparation of the TEM samples using the focused ion beam system.

Figure 7a presents a zebra crossing-like SEM image of a line array composed of the TiN/GSTC film stack fabricated by the SRAF-lithography and the subsequent etching, in which a uniform pitch line is confirmed in a size of up to around 7.4 × 6.9 μm^2^. The line width of around 70 nm across the array is achieved under the energy dose of 34 mJ/cm^2^. There are no typical defects, such as bridge, break, and line-collapse, identified in the array. The TEM images shown in Figure 7b–d present a uniform cross-sectional etching profile of the TiN/GSTC film stack across the array both in line width and space. The line at the edge of the array stands erect on the SiO_2_ layer with a width approximate to the rest of the lines. The appropriate over etching of SiO_2_ is realized by tuning the etching parameters, in which the TiN/GSTC film stack is fully separated. The etching damage on the phase change material is minimized as shown in the scanning TEM (STEM) and EDS mapping of Figure 8. The elemental mappings of Ge, Sb, Te, Ti, and N atoms suggest a uniform composition distribution without atomic diffusion detected in the film stack.

## 4. Conclusions

In this work, we demonstrate the influence of OPE in the lithography and micro-loading effect in the plasma etching on the morphology of phase change material arrays. The SRAF with the selected configuration is found to be highly effective at minimizing the OPE in the lithography process to improve the uniformity of the line width, especially for the edge line of the array. The various 70 nm half-pitch arrays on the 12-inch wafer were fabricated successfully by the placement of the SRAF. The combination of the SRAF-lithography and optimized plasma etching demonstrate a high uniformity, both in the profile of the line array and the elemental distribution of the phase change material. The research provides a path for the manufacturing of memory arrays with high density and outstanding reliability for the storage class memory applications.

## Data Availability

The data that support the findings of this study are available from the corresponding authors upon reasonable request.
